# Insurance Status and Emergency Department Visits Associated With Hemodialysis in Texas

**DOI:** 10.1001/jamanetworkopen.2019.21447

**Published:** 2020-02-19

**Authors:** Julianna West, Hei Kit Chan, Donald A. Molony, David J. Robinson, John R. Foringer, Henry E. Wang

**Affiliations:** 1 Department of Emergency Medicine, McGovern Medical School, The University of Texas Health Science Center at Houston

## Abstract

This cross-sectional study analyzes emergency department visits associated with hemodialysis by insured and uninsured patients in Texas.

## Introduction

Patients with end-stage renal disease (ESRD) require regular hemodialysis (HD) treatment, which is nearly universally covered by Medicare in the United States.^1^ However, Medicare coverage is not available for individuals who are not US citizens or permanent residents. For many uninsured individuals with ESRD, intermittent dialysis through the emergency department (ED) is the sole treatment option.^1^ The system of accessing HD in the ED presents several major system-level challenges, including adding patient volume to overcrowded EDs, taxing hospital dialysis resources, and incurring substantial health care costs. Prior studies^1,2^ characterizing the health burden of ED visits for HD treatment by patients with ESRD were limited to a single region. We sought to characterize ED visits for HD by insured and uninsured patients in Texas.

## Methods

We performed a cross-sectional analysis using the 2017 Texas Emergency Department Data Set. The Committee for the Protection of Human Subjects at The University of Texas Health Science Center at Houston approved the study and waived the need for informed consent because we used a preexisting set of deidentified data.

We included all ED visits by patients aged 18 years or older. We identified ED visits for HD treatment according to *International Statistical Classification of Diseases and Related Health Problems, Tenth Revision* codes 5A1D00Z and 5A1D60Z to 5A1D90Z and *Healthcare Common Procedure Coding System*/*Current Procedural Terminology* codes G0257, 90935, 90937, 90957 to 90970, and 90999.^3^ To identify instances where the ED visit was most likely associated with HD for an acute indication, we limited the analysis to hospitalizations with a length of stay of 1 day or less. The primary exposure was insurance status. We classified Medicare, Medicaid, and commercial insurance as insured, and classified self-pay, charity, indigent, or unknown as uninsured. We determined the total number of ED visits for HD treatment, stratifying by insurance status. We converted reported hospital charges to estimated costs by using Healthcare Cost Utilization Project cost-to-charge ratios.^4^ We identified differences between insured and uninsured individuals using logistic regression analysis. All analyses were performed using Stata statistical software version 15.0 (StataCorp). Data analysis was performed from May 2019 to January 2020.

## Results

There were a total of 8 392 693 adult ED visits; 6 968 438 visits had a length of stay of 1 day or less. Of all adult patients who visited the ED, 9786 (48.3%) were aged 18 to 44 years and 15 355 (60.9%) were female. Hemodialysis was associated with 33 829 ED visits, including 10 390 uninsured patients (incidence, 1.24 cases per 1000 adult ED visits; 95% CI, 1.22-1.26 cases per 1000 adult ED visits) and 23 439 insured patients (Figure). Most uninsured ED visits for HD originated from the Arlington (region 2/3; 6867 visits [66.1%]) and Houston (region 6/5S; 2123 visits [20.4%]) regions (Table).^5^ Uninsured patients requiring HD were more likely than insured patients to be younger (aged 18-44 years, 4158 [40.0%] vs 5628 [24.0%]), have white race (6870 [66.1%] vs 10 524 [44.9%]), and have Hispanic ethnicity (8893 [85.6%] vs 12 668 [54.0%]). There were no significant sex differences. Most patients requiring HD were discharged to home or home health care. Total hospital costs for uninsured HD visits were $21 837 047.40.

**Figure.**
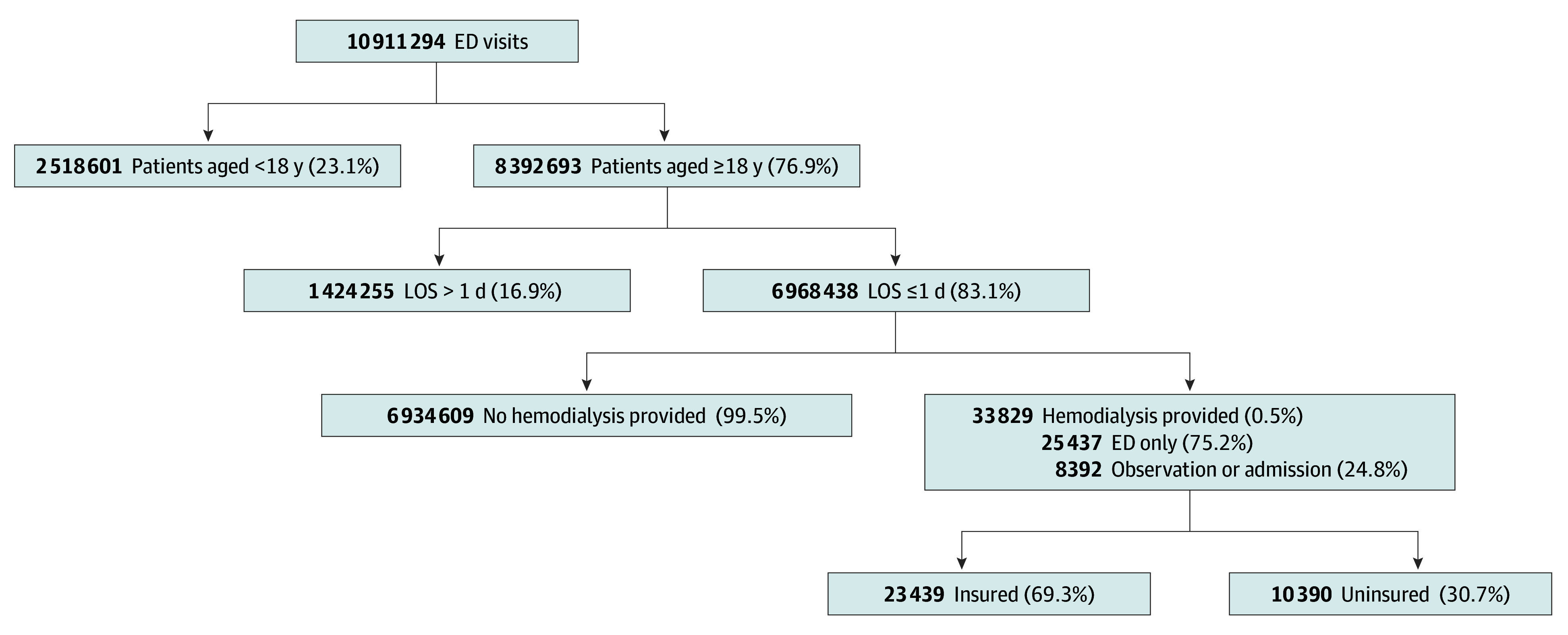
Emergency Department (ED) Visits Associated With Hemodialysis LOS indicates length of stay.

**Table.** Characteristics of Patients Who Made Emergency Department Visits for Hemodialysis, Stratified by Insurance Status

Characteristic	Patients, No. (%)		OR (95% CI)^b^
	Uninsured (n = 10 390)^a^	Insured (n = 23 439)	
Age range, y			
18-44	4158 (40.0)	5628 (24.0)	1 [Reference]
45-64	4758 (45.8)	11 106 (47.4)	0.58 (0.55-0.61)
65-74	1142 (11.0)	4585 (19.6)	0.34 (0.31-0.37)
≥75	331 (3.2)	2120 (9.0)	0.21 (0.19-0.24)
Unknown	1 (0.0)	0	NA
Sex			
Male	5355 (51.5)	11 818 (50.4)	1 [Reference]
Female	4865 (46.8)	10 490 (44.8)	0.98 (0.93-1.02)
Unknown	170 (1.6)	1131 (4.8)	NA
Race			
White	6870 (66.1)	10 524 (44.9)	1 [Reference]
American Indian, Eskimo, or Aleut	5 (0.1)	29 (0.1)	0.26 (0.10-0.68)
Asian or Pacific Islander	178 (1.7)	381 (1.6)	0.72 (0.60-0.86)
Black	511 (4.9)	5553 (23.7)	0.14 (0.13-0.16)
Other	2826 (27.2)	6952 (29.7)	0.62 (0.59-0.66)
Ethnicity			
Hispanic origin	8893 (85.6)	12 668 (54.0)	1 [Reference]
Not of Hispanic origin	1495 (14.4)	10 756 (45.9)	0.20 (0.19-0.21)
Unknown	2 (0.0)	15 (0.1)	NA
Principal diagnosis^c^			
Fluid overload (E87.7-E87.9)	4865 (46.8)	6359 (27.1)	1 [Reference]
Hyperkalemia (E87.5)	2011 (19.4)	3017 (12.9)	0.87 (0.81-0.93)
Hypertensive chronic kidney disease (I12.0-I13.2)	1870 (18.0)	5041 (21.5)	0.48 (0.45-0.52)
Diabetes with kidney complications (E10.2-E11.29)	504 (4.9)	645 (2.8)	1.02 (0.90-1.15)
End-stage renal disease (N18.5-N18.6)	377 (3.6)	363 (1.6)	1.36 (1.17-1.58)
Dyspnea (R060.0-R06.09)	90 (0.9)	371 (1.6)	0.32 (0.25-0.40)
Complications of cardiac and vascular devices and procedures^d^	64 (0.6)	872 (3.7)	0.10 (0.07-0.12)
Chest pain (R07.1-R07.9)	55 (0.5)	795 (3.4)	0.09 (0.07-0.12)
Respiratory failure (J96.0-J96.92)	46 (0.4)	260 (1.1)	0.23 (0.17-0.32)
Hypertensive crisis (I16.0-I16.9)	37 (0.4)	357 (1.5)	0.14 (0.10-0.19)
Infection^e^	35 (0.3)	630 (2.7)	0.07 (0.05-0.10)
Other	436 (4.2)	4729 (20.2)	0.12 (0.11-0.13)
Discharge status			
Discharged to home or home health care	10 189 (98.1)	21 018 (89.7)	1 [Reference]
Discharged or transferred to other health care facility	12 (0.1)	142 (0.6)	0.17 (0.10-0.31)
Discharged to skilled nursing, hospice, or swing bed	14 (0.1)	695 (3.0)	0.04 (0.02-0.07)
Left against medical advice	110 (1.1)	955 (4.1)	0.24 (0.19-0.29)
Died	30 (0.3)	286 (1.2)	0.22 (0.15-0.32)
Unknown or other	34 (0.3)	343 (1.5)	NA
Texas Public Health Region			
1: Lubbock			
Patients, No. (%)	7 (0.1)	318 (1.4)	1 [Reference]
Incidence/10 000 population (95% CI)^f^	0.1 (0.0-0.2)	3.7 (3.3-4.1)	
2/3: Arlington (Dallas–Fort Worth)			
Patients, No. (%)	6867 (66.1)	6625 (28.3)	47.09 (22.25-99.65)
Incidence/10 000 population (95% CI)^f^	9.0 (8.8-9.2)	8.7 (8.4-8.9)	
4/5N: Tyler			
Patients, No. (%)	81 (0.8)	641 (2.7)	5.74 (2.62-12.57)
Incidence/10 000 population (95% CI)^f^	0.5 (0.4-0.7)	4.2 (3.9-4.6)	
6/5S: Houston			
Patients, No. (%)	2123 (20.4)	8599 (36.7)	11.21 (5.30-23.75)
Incidence/10 000 population (95% CI)^f^	3.1 (3.0-3.2)	12.5 (12.3-12.8)	
7: Temple (Austin)			
Patients, No. (%)	131 (1.3)	972 (4.1)	6.12 (2.83-13.23)
Incidence/10 000 population (95% CI)^f^	0.4 (0.3-0.5)	3.1 (2.9-3.3)	
8: San Antonio			
Patients, No. (%)	333 (3.2)	1038 (4.4)	14.57 (6.82-31.13)
Incidence/10 000 population (95% CI)^f^	1.2 (1.1-1.3)	3.8 (3.6-4.0)	
9/10: El Paso			
Patients, No. (%)	66 (0.6)	563 (2.4)	5.33 (2.41-11.75)
Incidence/10 000 population (95% CI)^f^	0.5 (0.3-0.6)	3.8 (3.5-4.2)	
11: Harlingen (Laredo/Brownsville)			
Patients, No. (%)	744 (7.2)	4366 (18.6)	7.74 (3.64-16.44)
Incidence/10 000 population (95% CI)^f^	3.4 (3.1-3.6)	19.9 (19.3-20.5)	
Unknown, patients, No. (%)	38 (0.4)	317 (1.4)	NA
Hospital cost, $			Difference
Per patient, mean (95% CI)	2101.74 (2074.00-2129.47)	3238.70 (3202.94-3274.47)	1136.97 (1080.16-1193.77)
Total across state, millions	21.8	75.9	54.0

Abbreviations: NA, not applicable; OR, odds ratio.

^a^ Not all percentages sum to 100% because of rounding.

^b^ Odds ratios reflect differences in insured and uninsured. For example, patients in the age group 45 to 64 years are 0.58 times less likely to be uninsured than insured than patients in the age group 18 to 44 years.

^c^ *International Statistical Classification of Diseases, Tenth Revision, Clinical Modification *codes are shown for each diagnosis.

^d^ *International Statistical Classification of Diseases, Tenth Revision, Clinical Modification *codes T81.7 and T82.

^e^ *International Statistical Classification of Diseases, Tenth Revision, Clinical Modification *codes A0 to A4, B3 to B4, B9, G0, H0, H4, T3, J0 to J1, J4, K0 to K1, K3, K5 to K8, N10 to N16, N3 to N5, N7, O0, O2, O8, O9, O4, L03, L05, M00 to M02, M8-M9, and T7 to T8.

^f^ Incidence rate is calculated by dividing *n* by the Texas Department of State Health Services 2013 population estimates for each public health region.

## Discussion

We identified 10 390 ED visits for HD by uninsured patients in Texas in 2017. These ED visits resulted in more than $21.8 million in hospital costs. To our knowledge, these are the largest estimates of the statewide burden of uninsured HD.^1,2^ Regardless of some limitations, including the possibility of a regular HD source for these patients, our findings highlight the total number of HD-associated ED visits for which payment was not expected. In addition to increasing patient numbers and cost, uninsured HD-associated ED visits cause health care system strain because the determination of the need for HD often requires additional diagnostic tests, and individuals with ESRD not undergoing regular HD often present in clinical crisis,^1^ requiring significant inpatient stabilizing care in addition to HD. The observed regional variations in uninsured HD visits underscores the need for solutions that reflect differences in local populations and health resources. A visit to the ED for HD costs approximately $2000, as opposed to $250 for a scheduled outpatient session.^6^ Strategies such as providing scheduled outpatient HD for uninsured patients with ESRD^1^ and treating insured patients in after-hours outpatient settings could be a cost-effective alternative to ED visits for HD, potentially alleviating system burdens, saving health care resources, and achieving improved patient outcomes.
